# Successful trans‐septal ablation of a left concealed accessory pathway in a patient receiving surgical mitral valve repair and mechanical aortic valve replacement

**DOI:** 10.1111/anec.12808

**Published:** 2020-10-18

**Authors:** Ting‐Chun Huang, Jing‐Hsiung Tsai, Ju‐Yi Chen

**Affiliations:** ^1^ Division of Cardiology Department of Internal Medicine College of Medicine National Cheng Kung University Hospital National Cheng Kung University Tainan Taiwan

**Keywords:** ablation, atrioventricular reentrant tachycardia, mitral annuloplasty

## Abstract

We presented a case of severe aortic regurgitation and moderate mitral regurgitation s/p aortic valve replacement and mitral valve repair. Deterioration of tachyarrhythmia attacks was noted. In EP study, left lateral accessory pathway with orthodromic atrioventricular reentrant tachycardia was identified. We successfully ablated the accessory pathway by trans‐septal approach. Even though trans‐septal approach currently is a daily routine of invasive interventional electrophysiologists, in this case, we want to emphasize and illustrate the distance between true mitral annulus and coronary sinus. Unrecognizing this concept could result in efficacy and safety of catheter‐based therapy.

## 1 CASE PRESENTATION

**FIGURE 1:**
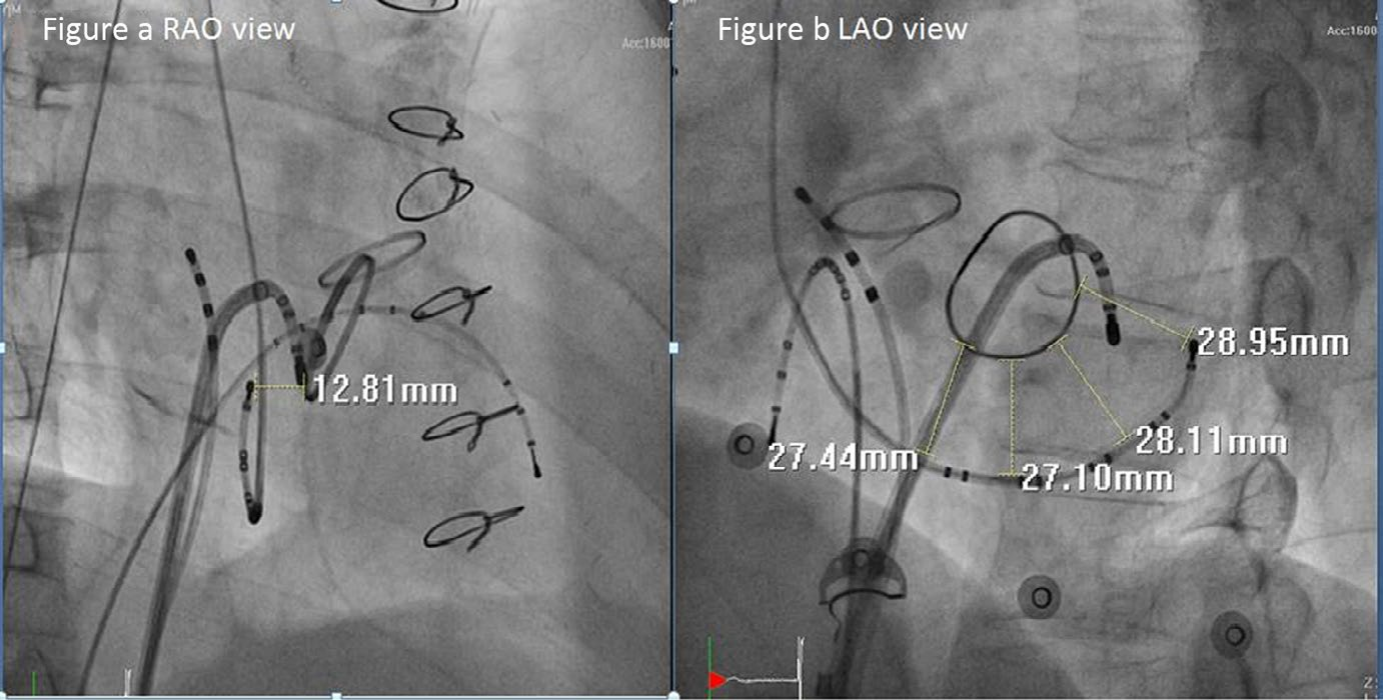
(a) Right anterior oblique (RAO) view and (b) left anterior oblique (LAO) view of the anatomical relationship among CS lead, artificial mitral annular ring, and ablation catheter. The CS lead was superior to the true mitral annulus (MA), and represented posterior left atrial wall. The CS to MA distance is measured as 12.81 mm in RAO view and as 27.9 mm in average at LAO view

**FIGURE 2:**
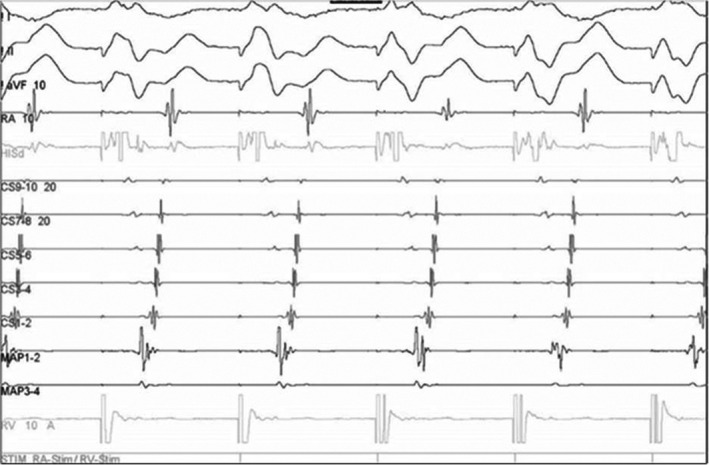
The local electrogram of the ablation catheter (MAP1‐2) showed ventriculoatrial fusion (the shortest stimulation‐to‐A interval) at the left lateral mitral ring, with an atrioventricular ratio of 1:3

A 60‐year‐old male patient was seen with a complaint of frequent palpitations. His medical history was significant for severe aortic regurgitation and moderate mitral regurgitation. He had received mitral valve annuloplasty with a 28 mm ring. He also received mechanical aortic valve replacement 2 years ago. He had visited emergency department several times for adenosine sensitive tachycardia after successful operation for valvular heart disease. The surface 12‐lead ECG showed narrow QRS tachycardia (167 beats per minute) with obscure retrograde P waves in the inferior leads. Electrophysiological studies showed that atrial extra‐stimulus induced orthodromic atrioventricular reentrant tachycardia (AVRT). Due to mechanical aortic valve, we used antegrade trans‐septal approach. Subvalvular areas were examined to avoid damaging the mitral annuloplasty ring (Figure 1a,b). A local electrogram (EGM) under RV pacing showed ventriculoatrial (VA) fusion (the shortest stimulation‐to‐A interval) at the left lateral mitral ring, with an atrioventricular ratio of 1:3 (Figure 2). Immediately after energy application, complete VA block was achieved.

## 2 DISCUSSION

In this case report, two major concepts should be emphasized: first, the anatomical relationship of coronary sinus to true mitral annulus and, second, the feasibility of ablation for left side accessory pathway in patients who had even undergone mitral annuloplasty.

Tops et al. (2007) used 64‐slice multi‐slice computed tomography to evaluate the anatomical relationship between the coronary sinus and mitral annulus, and found that coronary sinus (CS) is superior to the mitral annulus (MA), and the minimal distance was 5.1 ± 2.9 mm. When patients had more severe MR, the minimal distance was significantly greater. In this case, we clearly illustrate that the CS lead represented the posterior left atrial wall rather than the true mitral annulus. The reliance on CS leads could lead to misunderstanding of the true anatomy.

Several groups (Choure et al., 2006; Kim et al., 2016; Shinbane et al., 1998) measured the CS to MA distance by different methods, such as left fluoroscopic atriogram, multi‐detector computed tomography, and even cadaver human heart. Since there was no uniform methodology, it makes the distance differ and thereby making the direct comparisons of these studies difficult. Moreover, the CS diameter is inconsistent. One study (Shah et al., 2012) used contrast‐enhanced non‐ECG‐gated chest computed tomography, and the mean diameter of the CS as reported to be 7.05 ± 1.90 mm. Lansac et al. (2008) studied CS of cadaver human hearts, and it was away from the native MA around 13.7 to 20.4 mm at the middle site. Our patient's CS to MA distance may be within the range of 18.95–22.75 mm after eliminating the CS diameter influence. The distance between CS and MA in our patient is different from other case reports. Underlying severe mitral regurgitation‐related left atrial dilatation, bottom CS lead position via trans‐groin approach, and different angles of fluoroscopy all would influence the measurement.

Surgical treatment of accessory pathways and concomitant valvular heart diseases was suggested before the era of radiofrequency ablation (Cox & Ferguson, 1989; Misaki et al., 1994), but our patient developed symptoms after surgery. The artificial ring is implanted endocardially and impeded ablations for accessory pathways which pass on the epicardial aspect of the annulus fibrosus (Becker & Anderson, 1981). Although the ventricular insertion usually branches into multiple connections with the ventricle, searching and ablating the ventricular insertions by RV pacing could avoid damaging the artificial ring and provided an effective treatment for accessory pathway ablation (Yamabe et al., 2002).

## CONFLICTS OF INTEREST

The authors declare that they have no conflicts of interest with the content of this case report.

## AUTHOR CONTRIBUTIONS

All authors reviewed and approved the manuscript. Ting‐Chun Huang, manuscript writing, data collection, and idea conceptualization; Jing‐Hsiung Tsai, data collection; Ju‐Yi Chen, idea conceptualization, gave suggestions on this manuscript, final approval.

## ETHICS

This case report was writing respecting patient confidentiality and privacy. Patient had the opportunity to read the present case report and had no objections to the final abstract.

## References

[anec12808-bib-0001] Becker, A. E. , & Anderson, R. H. (1981). The Wolff‐Parkinson‐White syndrome and its anatomical substrates. Anatomical Record, 201, 169–177. 10.1002/ar.1092010118 7305018

[anec12808-bib-0002] Choure, A. J. , Garcia, M. J. , Hesse, B. , Sevensma, M. , Maly, G. , Greenberg, N. L. , Borzi, L. , Ellis, S. , Tuzcu, E. M. , & Kapadia, S. R. (2006). In vivo analysis of the anatomical relationship of coronary sinus to mitral annulus and left circumflex coronary artery using cardiac multidetector computed tomography: Implications for percutaneous coronary sinus mitral annuloplasty. Journal of the American College of Cardiology, 48(10), 1938–1945. 10.1016/j.jacc.2006.07.043 17112981

[anec12808-bib-0003] Cox, J. L. , & Ferguson, T. B. Jr (1989). Surgery for the Wolff‐Parkinson‐White syndrome: The endocardial approach. Seminars in Thoracic and Cardiovascular Surgery, 1, 34–46. 2488406

[anec12808-bib-0004] Kim, J.‐W. , Hwang, G.‐S. , Seo, K.‐W. , Park, J.‐S. , Yang, H.‐M. , Lim, H.‐S. , Choi, B.‐J. , Choi, S.‐Y. , Yoon, M.‐H. , & Tahk, S.‐J. (2016). Anatomical discrepancy between the coronary sinus and the mitral annulus by fluoroscopy. International Journal of Arrhythmia, 17(1), 14–19. 10.18501/arrhythmia.2016.002

[anec12808-bib-0005] Lansac, E. , Di Centa, I. , Al Attar, N. , Messika‐Zeitoun, D. , Raffoul, R. , Vahanian, A. , & Nataf, P. (2008). Percutaneous mitral annuloplasty through the coronary sinus: An anatomic point of view. Journal of Thoracic and Cardiovascular Surgery, 135(2), 376–381. 10.1016/j.jtcvs.2007.05.071 18242272

[anec12808-bib-0006] Misaki, T. , Watanabe, G. , Iwa, T. , Matsunaga, Y. , Ohotake, H. , Tsubota, M. , Yamamoto, K. , & Watanabe, Y. (1994). Surgical treatment of patients with Wolff‐Parkinson‐White syndrome and associated acquired valvular heart disease. Journal of Thoracic and Cardiovascular Surgery, 108, 68–72. 10.1016/S0022-5223(94)70220-9 8028381

[anec12808-bib-0007] Shah, S. S. , Teague, S. D. , Lu, J. C. , Dorfman, A. L. , Kazerooni, E. A. , & Agarwal, P. P. (2012). Imaging of the coronary sinus: Normal anatomy and congenital abnormalities. Radiographics, 32(4), 991–1008. 10.1148/rg.324105220 22786990

[anec12808-bib-0008] Shinbane, J. S. , Lesh, M. D. , Stevenson, W. G. , Klitzner, T. S. , Natterson, P. D. , Wiener, I. , Ursell, P. C. , & Saxon, L. A. (1998). Anatomic and electrophysiologic relation between the coronary sinus and mitral annulus: Implications for ablation of left‐sided accessory pathways. American Heart Journal, 135(1), 93–98. 10.1016/s0002-8703(98)70348-5 9453527

[anec12808-bib-0009] Tops, L. F. , Van de Veire, N. R. , Schuijf, J. D. , de Roos, A. , van der Wall, E. E. , Schalij, M. J. , & Bax, J. J. (2007). Noninvasive evaluation of coronary sinus anatomy and its relation to the mitral valve annulus: Implications for percutaneous mitral annuloplasty. Circulation, 115, 1426–1432. 10.1161/CIRCULATIONAHA.106.677880 17353434

[anec12808-bib-0010] Yamabe, H. , Shimasaki, Y. , Honda, O. , Kimura, Y. , & Hokamura, Y. (2002). Localization of the ventricular insertion site of concealed left‐sided accessory pathways using ventricular pace mapping. Pacing and Clinical Electrophysiology, 25, 940–950. 10.1046/j.1460-9592.2002.t01-7-00940.x 12137347

